# A Novel *α*-L-Arabinofuranosidase of Family 43 Glycoside Hydrolase (*Ct*43Ara*f*) from *Clostridium thermocellum*

**DOI:** 10.1371/journal.pone.0073575

**Published:** 2013-09-09

**Authors:** Shadab Ahmed, Ana Sofia Luis, Joana L. A. Bras, Arabinda Ghosh, Saurabh Gautam, Munishwar N. Gupta, Carlos M. G. A. Fontes, Arun Goyal

**Affiliations:** 1 Department of Biotechnology, Indian Institute of Technology Guwahati, Guwahati, Assam, India; 2 Department of Chemistry, Indian Institute of Technology Delhi, Hauz Khas, New Delhi, India; 3 CIISA-Faculdade de MedicinaVeterinaria, Avenida da Universidade Técnica, Lisbon, Portugal; University of Cantebury, New Zealand

## Abstract

The study describes a comparative analysis of biochemical, structural and functional properties of two recombinant derivatives from *Clostridium thermocellum* ATCC 27405 belonging to family 43 glycoside hydrolase. The family 43 glycoside hydrolase encoding α-L-arabinofuranosidase (*Ct*43Ara*f*) displayed an N-terminal catalytic module *Ct*GH43 (903 bp) followed by two carbohydrate binding modules *Ct*CBM6A (405 bp) and *Ct*CBM6B (402 bp) towards the C-terminal. *Ct*43Ara*f* and its truncated derivative *Ct*GH43 were cloned in pET-vectors, expressed in *Escherichia coli* and functionally characterized. The recombinant proteins displayed molecular sizes of 63 kDa (*Ct*43Ara*f*) and 34 kDa (*Ct*GH43) on SDS-PAGE analysis. *Ct*43Ara*f* and *Ct*GH43 showed optimal enzyme activities at pH 5.7 and 5.4 and the optimal temperature for both was 50°C. *Ct*43Ara*f* and *Ct*GH43 showed maximum activity with rye arabinoxylan 4.7 Umg^−1^ and 5.0 Umg^−1^, respectively, which increased by more than 2-fold in presence of Ca^2+^ and Mg^2+^ salts. This indicated that the presence of CBMs (*Ct*CBM6A and *Ct*CBM6B) did not have any effect on the enzyme activity. The thin layer chromatography and high pressure anion exchange chromatography analysis of *Ct*43Ara*f* hydrolysed arabinoxylans (rye and wheat) and oat spelt xylan confirmed the release of L-arabinose. This is the first report of *α*-L-arabinofuranosidase from *C. thermocellum* having the capacity to degrade both *p*-nitrophenol-*α*-L-arabinofuranoside and *p*-nitrophenol-*α*-L-arabinopyranoside. The protein melting curves of *Ct*43Ara*f* and *Ct*GH43 demonstrated that *Ct*GH43 and CBMs melt independently. The presence of Ca^2+^ ions imparted thermal stability to both the enzymes. The circular dichroism analysis of *Ct*GH43 showed 48% β-sheets, 49% random coils but only 3% α-helices.

## Introduction

Plant cell wall is mainly composed of complex structural polysaccharides like cellulose and hemicellulose [1], [2]. The hetero-polymers of pentoses like D-xylose, L-arabinose and hexoses *viz*. D-mannose, D-glucose and D-galactose constitutes the hemicellulose. Often, xylans are hetero-polysaccharides with 1,4-linked-β-D-xylopyranose backbone chains containing arabinose, glucuronic acid, or its 4-O-methyl ether, acetic, ferulic, and p-coumaric acids side chains depending mainly on the source of xylans [3]. Rye arabinoxylans contain arabinose and xylose in the A/X ratio of 0.49–0.82 and also ferulate residues attached to arabinose as esters at its O-5 position [4] but in wheat arabinoxylans the arabinose to xylose ratios [A/X] varies from 0.47 to 0.58 [5]. The L-arabinosyl residues are often found in hemicelluloses, such as arabinan, arabinoxylan, gum arabic and arabinogalactan. The cereal arabinoxylans are composed majorly of a backbone of 1,4-linked-β-D-xylopyranosyl residues substituted with single *α*-arabinofuranosyl substituents attached to the O-2, O-3 or to both O-2,3 of the xylose residues [6], [7]. It has been documented that *α*-L-arabinofuranosyl and to a lesser extent *α*-L-arabinopyranosyl side chains are attached to the β-D-galactopyranose main chain by 1,3- and 1,6- linkages in type II arabinogalactans [4], [6], [8], [9], [10]. The *α*-L-arabinofuranosidase (EC 3.2.1.55) are enzymes known to release terminal *α*-1,2-, *α*-1,3- and *α*-1,5 *α*-L-arabinofuranosyl residues from hemicellulose such as arabinoxylan and other L-arabinose containing polysaccharides [6], [9]. Arabinofuranosidase have been reported from a few glycoside hydrolase families (GHs) *viz*., GH30 [11], GH43 [12], [13], GH51 [14], [15], [16], GH54 [17] and GH62 [18], [19]. The GH43 arabinoxylan arabinofuranohydrolase from *Bacillus subtilis* reported by Bourgois *et al*., 2007 [12] specifically released arabinofuranosyl residues from the mono-substituted C-(O)-2 and C-(O)-3 xylopyranosyl residues on the xylan backbone. Whereas, Cartmell *et el*., 2011 [13] reported an arabinan-specific GH43 (α-1,2-arabinofuranosidase) from *Cellvibrio japonicus* capable of cleaving the α-1,2-arabinofuranose decorations in single or double substitutions. The arabinofuranosidase (Arf51B) from *Clostridium stercorarium* as reported by Aldesberger *et al*., (2004) was able to release α-L-arabinose residues from de-esterified arabinoxylan [15]. The arabinofuranosidase (Araf51) from *Clostridium thermocellum* as described by Taylor *et al*., (2006) catalyzed the hydrolysis of both α-1,5-linked arabino-oligosaccharides and the α-1,3 arabinosyl side chain of xylan with equal efficiency [16]. *α*-L-Arabinofuranosidases have been used synergistically with other cellulose degrading enzymes in agro-industrial processes [6], [9]. Lignocelluloses are known to have cellulose as major content but they also contain 20% hemicellulose which mainly consists of pentose such as xylose and arabinose [1], [2]. In the past *α*-L-arabinofuranosidases received less importance in the production of bio-ethanol because pentoses are less efficiently converted to ethanol than hexose sugars [20]. But recently they have been used along with *Candida shehatae* that utilizes the pentose sugars for bio-ethanol production from cellulosic waste like mango and poplar leaves [21]. Also L-arabinose has been shown to inhibit intestinal sucrase and reduce the glycaemic response after sucrose ingestion in animals [22]. The carbohydrate binding modules (CBMs) are the non-catalytic modules known to help or bring the catalytic modules in close proximity to its substrates and also some CBMs are known to stabilize the enzyme structure and increase its temperature resistance [23], [24]. The CBMs may be found to contain up to 200 amino acids and can be found attached as single, double or triple domains in one protein, located at both C- or N-terminal within the parental protein [23], [24].

Biochemical, structural and functional characterization of *Ct*43Ara*f* and *Ct*GH43 is essential as all the *α*-L-arabinofuranosidases have the same inverting mechanism of catalysis but the enzyme activities are different (http://www.cazy.org/GH43.html). In the present study the recombinant proteins *Ct*43Ara*f* and its truncated derivative *Ct*GH43 were investigated and biochemically, structurally and functionally characterized. To our knowledge this is the first report of any *α*-L-arabinofuranosidase from *Clostridium thermocellum* showing the ability to hydrolyze both 4-nitrophenyl-*α*-arabinofuranoside (*p*NPA*f*) and 4-nitrophenyl-*α*-arabinopyranoside (*p*NPA*p*).

## Materials and Methods

### Bacterial Strains and Plasmid

The genomic DNA of *Clostridium thermocellum* was a gift from Professor Carlos Fontes, Faculdade de MedicinaVeterinaria, Lisbon, Portugal. *Escherichia coli* (DH5α) cells were used for cloning, whereas, *E. coli* BL-21 (DE3) and *E. coli* BL-21 (DE3) pLysS cells were used as expression host. The plasmids used for cloning and expression were pET-21a(+) and pET-28a(+). All the above mentioned items were procured from Novagen (Madison, USA).

### Fine Chemicals, Natural and Synthetic Substrates for Enzyme Assay

Thin layer chromatography (TLC) and high pressure anion exchange chromatography (HPAEC) standards like xylose, L-arabinose, cellobiose, chelating agent *viz.,* EGTA and NaOH solution (50%, w/v) were procured from Sigma-Aldrich Chemicals Co., USA. Rye arabinoxylan, wheat arabinoxylan (soluble and insoluble), arabinogalactan, sugar beet arabinan, rhamnogalactouronan, 1,5-α-L-arabinobiose and 1,5-α-L-arabinotetraose were procured from Megazyme International, Ireland. Oat spelt xylan, birchwood xylan, beechwood xylan, β-D-glucan, carboxy methylcellulose (CMC), carboxy ethylcellulose (CEC) and synthetic *p*NP-glycosides like *p*NP-α-L-arabinofuranoside (*p*NPA*f*) and *p*NP- α-L-arabinopyranoside (*p*NPA*p*), were purchased from Sigma-Aldrich Chemicals Co., USA.

### Gene Amplification and Cloning

Oligonucleotide primers containing *Nhe*I and *Xho*I restriction sites were designed and the DNA encoding *Ct*43Ara*f* (GenBank Accession No: ABN52503.1; comprising of *Ct*GH43, *Ct*CBM6A and *Ct*CBM6B domains, sequence range 1545911 to 1547651) and truncated gene *Ct*GH43 were amplified from genomic DNA of *C. thermocellum* (7.7 ng) using 2.5 U of *Pfu* DNA polymerase (Stratagene, USA). A 50 µl PCR reaction mixture contained Mg^2+^ ions (2.5 mM), dNTPs (1.6 mM) forward and reverse primer (0.45 µM) and PCR-grade water (Sigma, USA). The primers used for amplifying *Ct*43Ara*f* were forward 5′-ctc**gctagc**gctgccgattatccg-3′, reverse 5′-cac**ctcgag**aattatgccactactgc-3′. Primers for *Ct*GH43 have been reported previously [21]. The PCR amplification cycles used were: denaturation at 94°C for 4 min followed by 30 cycles of i) denaturation at 94°C for 30 s, ii) annealing at 55°C for 60 s and iii) extension at 72°C for 2 min followed by a final extension at 72°C for 10 min. The PCR amplified DNA of *Ct*43Ara*f* was cloned in *Nhe*I*-Xho*I digested pET-21a(+) expression vector while PCR DNA of *Ct*GH43 was cloned earlier in pET-28a(+) vector [21], resulting in respective cloned plasmids. The positive clones were confirmed by *Nhe*I-*Xho*I digestion and DNA sequencing of recombinant plasmid. Thereafter, *E. coli* (DH5*α*) cells were transformed with above recombinant plasmids (p*Ct*43Ara*f* and p*Ct*GH43). The transformed cells were grown on LB-agar plates supplemented with ampicillin (100 µg ml^−1^) in case of *Ct*43Ara*f* and kanamycin (50 µg ml^−1^) in case of *Ct*GH43 at 37°C. Positive clones were screened by restriction digestion analysis of the isolated recombinant plasmids DNAs from the cells. The truncated enzyme *Ct*GH43 was amplified, cloned and expressed earlier and was reported [21].

### Expression and Purification of Recombinant α-L-arabinofuranosidase

*E. coli* BL-21 (DE3) pLysS cells were used for expressing *Ct*GH43 as described earlier [21], whereas, *E. coli* BL-21 (DE3) cells were transformed with recombinant plasmid p*Ct*43Ara*f*. The cells were grown in 100 ml LB medium containing ampicillin (100 µg ml^−1^) for growing *Ct*43Ara*f* and kanamycin (50 µg ml^−1^) for growing *Ct*GH43 at 37°C with 180 rpm till mid-exponential phase (A_550≈_0.6), the cells were then induced with isopropyl-1-thio-β-D-galactopyranoside (1.0 mM) for over-expression of recombinant proteins by incubation at 24°C with 180 rpm for 24 h [25]. The cells were harvested at 10,000 g at 4°C for 15 min using centrifuge (Sigma, 4 K 15) and the resulting cell pellets were re-suspended in 20 mM sodium phosphate buffer pH 7.4. Then the cells were sonicated (Sonics, Vibra cell) on ice for 8 min (5 s on/15 s off pulse; 33% amplitude) and again centrifuged at 19,000 g at 4°C for 30 min to get the cell free extract. The recombinant proteins from the cell free extracts were purified in a single step by immobilized metal ion affinity chromatography (IMAC) using sepharose columns (GE Healthcare, HiTrap chelating) and further dialyzed against 20 mM sodium phosphate buffer pH 5.7 for *Ct*43Ara*f* and 20 mM sodium acetate buffer pH 5.4 for *Ct*GH43 following the method as reported previously [21]. The purity and molecular mass of recombinant proteins were verified by SDS-PAGE [26].

### Substrate Specificity of *Ct*43Ara*f* and *Ct*GH43 against Natural Substrates

The enzyme assay for *Ct*43Ara*f* was performed using 20 mM sodium phosphate buffer (pH 5.7), whereas, the assay for *Ct*GH43 was carried out in 20 mM sodium acetate buffer (pH 5.4). The 100 µl reaction mixture contained 1.0%, w/v substrate, 10 µl of enzyme (*Ct*43Ara*f* 0.45 mg ml^−1^ or *Ct*GH43 0.5 mg ml^−1^) and in both cases the reaction mixture was incubated at 50°C for 15 min. ‘The enzyme activity was determined by measuring the reducing sugar by the method of Nelson and Somogyi [27], [28]. The concentration of reducing sugar was estimated using a standard curve of L-arabinose as both *Ct*43Ara*f* and *Ct*GH43 predominantly showed *α*-L-arabinofuranosidase activity. One unit of activity was defined as the amount of enzyme which produced 1 µmole of L-arabinose per min under the optimized condition of temperature and pH. For studying the pH profile of *Ct*43Ara*f*, 20 mM sodium acetate buffer, pH 4.0–5.6, 20 mM sodium phosphate buffer, pH 5.7–7.5, 20 mM Tris/HCl, pH 7.5–8.0, buffer were used in enzyme assays that employed 1.0 (%, w/v) rye arabinoxylan, similar to the method employed for optimization of *Ct*GH43 [21]. The optimum temperature and thermostability of *Ct*43Ara*f* was determined by performing assay at different temperatures following the method reported earlier [21]. The kinetic parameters of *Ct*43Ara*f* and *Ct*GH43 were determined by performing assays at varying concentrations of the soluble substrates such as rye arabinoxylan, wheat arabinoxylan, oat spelt xylan, beechwood xylan and birchwood xylan and insoluble wheat arabinoxylan under the optimized condition of temperature and pH. The optimum pH and pH stability profile was generated by performing the enzyme assays at the optimum temperature of 50°C. The experiments were performed in triplicate.

### Substrate Specificity of *Ct*43Ara*f* and *Ct*GH43 with Synthetic *p*-nitrophenyl-glycosides

The assays of *Ct*43Ara*f* and *Ct*GH43 with synthetic *p*-nitrophenyl glycosides (*p*NP-glycosides) *viz*., *p*-nitrophenyl-α-L-arabinofuranoside (*p*NPA*f*) and *p*-nitrophenyl-α-L-arabinopyranoside (*p*NPA*p*) were carried out by estimating the release of 4-nitrophenol (*p*NP) at 405 nm using a UV-Visible spectrophotometer (Varian, Cary 100 Bio) following the method described by Cartmell *et al*. 2011 [13]. The enzyme reaction was performed in 1.0 ml reaction mixture containing *p*NPA*f* or *p*NPA*p* in 50 mM sodium phosphate buffer (pH 6.0), 20 µl of enzyme (*Ct*43Ara*f* 0.45 mg ml^−1^ or *Ct*GH43 0.5 mg ml^−1^) incubated for 15 min at 50°C in a peltier temperature controller (Varian, Cary 100 Bio). The kinetic parameters of *Ct*43Ara*f* and *Ct*GH43 with *p*NPA*f* or *p*NPA*p* were determined by varying their concentrations in the range 20 to 500 µM. The initial (pseudo-first order) rates of *Ct*43Ara*f* and *Ct*GH43 with both the *p*NP-glycosides were measured in order to calculate the kinetic parameters. Continuous readings were recorded every one second for the initial linear absorbance range (0–15 mins) with an array of concentrations (20–500 µM) of *p*NP-glycosides. The reaction was stopped after 15 min by the addition 0.5 M sodium carbonate to make the reaction mixture highly alkaline (around pH 11.0**).** The assays were performed in triplicates. The released *p*-nitrophenol was quantified using the molar extinction coefficient of 24150 litre mole^−1^ cm^−1^ as reported by Cartmell *et al.* 2011 [13].

### Effects of Metal Ions and Chemical Agents

The effects of different metal cations on the activity of *Ct*43Ara*f* (0.45 mg ml^−1^) and *Ct*GH43 (0.5 mg ml^−1^) were determined using 100 µl reaction mixture (in duplicates) with oat spelt xylan (1%, w/v) as the substrate and adding respective metal salt at low molar concentrations (up to 20 mM). Assays for *Ct*43Ara*f* using 20 mM sodium phosphate buffer (pH 5.7) and for *Ct*GH43 using 20 mM sodium acetate buffer (pH 5.4) were performed at 50°C. The reaction mixtures in both the cases were subjected to 15 min of incubation. The blank with substrates having the respective salts were also assayed in parallel. The effects of various salts of Na^+^, Ca^2+^, Mg^2+^, Mn^2+^, Zn^2+^, Cu^2+^, Co^2+^, Ni^2+^, Al^3+^ and chelating agents like disodium EDTA and disodium EGTA were studied by varying their concentrations from 2.0–20.0 mM in the enzyme-substrate reaction mixture. The enzyme activity was calculated by estimating the reducing sugars as described above.

### Thin Llayer Chromatography of *Ct*43Ara*f* Hydrolyzed Products

The qualitative analysis of *Ct*43Ara*f* hydrolyzed products of natural substrates was performed by thin layer chromatography (TLC) on silica gel-coated aluminium plate (TLC Silica gel 60 F_254,_ 20×20 cm, Merck) for detecting the released sugars. The enzyme catalyzed reactions with 1% (w/v) substrate (rye arabinoxylan, wheat arabinoxylan or oat spelt xylan) were carried out in 100 µl reaction mixture maintaining the optimized condition of temperature and pH as mentioned above. The 100 µl reaction mixture was then precipitated with 2 volumes of acetone and centrifuged at 4°C at 13,000 g for 5 min [29]. The supernatant was transferred to another micro-centrifuge tube and the reaction product precipitate was concentrated by evaporating the acetone. Then 2.0 µl of sample (enzyme-substrate reaction product) as well as of standard (L-arabinose and D-xylose) solutions (1.0 mg ml^−1^) were loaded on the TLC plate. The plate was dried for few min and kept in the developing chamber saturated with the developing solution (mobile phase). The mobile phase consisted of acetic acid**-** *n*-propanol**-**water**-**acetonitrile in the ratio 4∶10:11∶14 [29]. At the end of the run, migrated sugars were visualized by immersing the chromatogram in a solution (sulphuric acid: methanol 5∶95, v/v; and *α*-napthol 5.0%, w/v). The TLC plates were then dried in a hot-air oven at 80°C for 20 min. The migrated sugars appeared as blue spots on the TLC plate.

### HPAEC Analysis of Polysaccharide Hydrolysis Product by *Ct*43Ara*f*

*Ct*43Ara*f* (4.7 U/mg, 0.5 mg/ml) catalyzed reactions with 1% (w/v) substrate (rye arabinoxylan, wheat arabinoxylan and oat spelt xylan) was carried out in 100 µl reaction mixture maintaining the optimal assay conditions as mentioned above. The 100 µl reaction mixture was incubated for 30 min at 50°C. The reaction was stopped by boiling the reaction mixture in a boiling water bath for 5 min. The 100 µl reaction mixtures were treated with 2 volumes of acetone to precipitate the remaining polysaccharides (substrates) and then centrifuged at 13,000 g for 10 min at 4°C. The supernatant containing the liberated sugar was transferred to another micro centrifuge tube and the acetone was removed by evaporation. The supernatant (50 µl) was diluted to 500 µl by adding ultra-pure (MilliQ) water and filtered through syringe filter using 0.2 µm membrane. The liberated sugars released due to the polysaccharide hydrolysis by enzyme reaction were analysed by high pressure anion exchange chromatography (HPAEC) using ion chromatography system (Dionex, ICS-3000). From the filtered 500 µl, 25 µl of sample (liberated sugars) was run on CARBOPACK™ PA-20 column (150×3 mm, Dionex), attached with CarboPac™ PA20 guard column (30×3 mm, Dionex) with Borate and Amino trap columns which removed impurities and provided high resolution. The instrument (Dionex, ICS-3000) was kept at constant temperature of 30°C during the analysis. The sample loop (sample loaded) size was kept to 25.0 µl and the flow rate was maintained at 0.3 ml min^−1^. The elution of liberated sugars released due to enzymatic reaction was carried out with 50.0 mM sodium hydroxide (Sigma, USA) using pulsed amperometric detector (PAD). L-arabinose and D-xylose (10 µg ml^−1^) were used as standards. The solutions of standards as well as samples were filtered by passing through 0.2 *µ*m filter before loading on the column.

### Protein Melting Study of *Ct*43Araf and *Ct*GH43

Protein melting curves were generated by subjecting recombinant proteins (*Ct*43Ara*f* and *Ct*GH43) to varying temperatures and measuring the change in the absorbance at 280 nm (tryptophan absorption maximum) by a UV-Visible spectrophotometer (Varian, Cary 100-Bio) following the method of Dvortsov *et al.* [30]. The column (IMAC) purified *Ct*43Ara*f* was dialyzed against 20 sodium phosphate buffer, pH 5.7, while, purified *Ct*GH43 was dialyzed against 20 mM sodium acetate buffer, pH 5.4. The protein concentration for both *Ct*43Ara*f* and *Ct*GH43 were kept at 0.4 mg ml^−1^. The absorbance at 280 nm was measured at different temperatures varying from 40–90°C using a peltier temperature controller. The protein solutions (1 ml) of *Ct*43Ara*f* and *Ct*GH43 were kept at the particular temperature for 10 min to attain the equilibrium. Similar experiment was carried out; with the addition of 10 mM CaCl_2_ in the 1 ml enzyme (0.4 mg/ml) solution and the temperature was then varied. The experiment was repeated with the addition of CaCl_2_ and EGTA to 1 ml enzyme solution (0.4 mg ml^−1^) in equimolar concentrations of 10 mM, and finally the change in absorbance at 280 nm was measured. The relative derivative absorption coefficient is the normalization of melting points calculated at each increasing temperature repeated twice with an error of 5%. The relative derivative absorption coefficients were calculated using the Agilent ChemStation for UV-Visible Spectroscopy software (Agilent Technologies, USA) as described by Dvortsov *et al.*, [30]. A curve of relative derivative absorption coefficient versus temperature was plotted to display the melting profile of *Ct*43Ara*f* and *Ct*GH43.

### Circular Dichroism Analysis of Truncated Catalytic Derivative *Ct*GH43

Far-UV Circular dichroism (CD) spectra of *Ct*GH43 were recorded on a spectropolarimeter (Jasco Corporation, Tokyo, JASCO J-815), equipped with a peltier system for temperature control at 25°C using a cell with a path length of 0.1 cm. The spectral accumulation parameters were carried out using a scan-rate of 50 nm min^−1^, a 1 nm bandwidth in the wavelength range of 195–250 nm with an average of six scans for each far-UV spectrum. The CD spectra of *Ct*GH43 is presented in terms of mean residue ellipticity (MRE, expressed as deg cm^2^ dmol^−1^) as a function of wavelength, calculated following the procedure described earlier [31] using a protein concentration of 15 µM in 10 mM Tris-HCl, pH 7.5. The CD spectra were corrected for buffer contributions and secondary structures were calculated by using web based K2d neural network software package (http://www.ogic.ca/projects/k2d2/) as described by Perez-Iratxeta and Andrade-Navarro [32].

## Results

### Gene Amplification and Cloning

**Figure 1:**
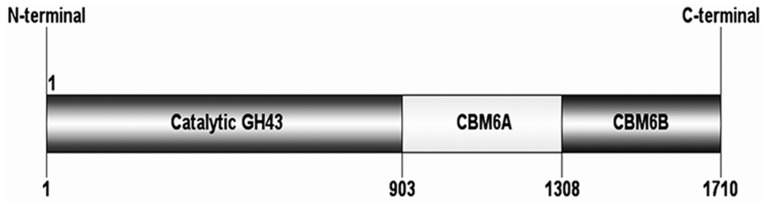
The molecular architecture of *Ct*43Ara*f* shows modular structure with an N-terminal family 43 glycoside hydrolase (*Ct*GH43) catalytic module (903 bp), a C-terminal family 6 carbohydrate binding module (*Ct*CBM6B, 402 bp) and another family 6 Carbohydrate binding module (*Ct*CBM6A, 405 bp) sandwiched between these two modules.

The molecular architecture of family 43 glycoside hydrolase (*Ct*43Ara*f*) from *C. thermocellum* displays an N-terminal family 43 glycoside hydrolase catalytic module (*Ct*GH43, 903 bp) followed by two successive carbohydrate binding modules (CBMs) *Ct*CBM6A (405 bp) and *Ct*CBM6B (402 bp) at the C-terminus [Fig. 1]. The DNA encoding *Ct*43Ara*f* (GenBank Accession No: ABN52503.1 comprising modules *Ct*GH43, *Ct*CBM6A and *Ct*CBM6B) and its truncated derivative (*Ct*GH43) were PCR-amplified using oligonucleotide primers containing *Nhe*I (**gctagc**) and *Xho*I (**ctcgag**) restriction sites. The PCR-amplified DNA of *Ct*43Araf was digested with *Nhe*I*-Xho*I restriction enzyme. The digested fragment of *Ct*43Ara*f* was cloned into *Nhe*I*-Xho*I digested pET-21a(+) expression-vector, while PCR amplified and digested DNA of *Ct*GH43 was cloned earlier in pET-28a(+) vector earlier [21], resulting in respective recombinant plasmids. The positive clones of *Ct*43Ara*f* and *Ct*GH43 were identified by restriction enzyme digestion. *Ct*GH43 was amplified, cloned and expressed earlier and reported [21].

### Expression and Purification of Recombinant *Ct*43Araf and *Ct*GH43

**Figure 2:**
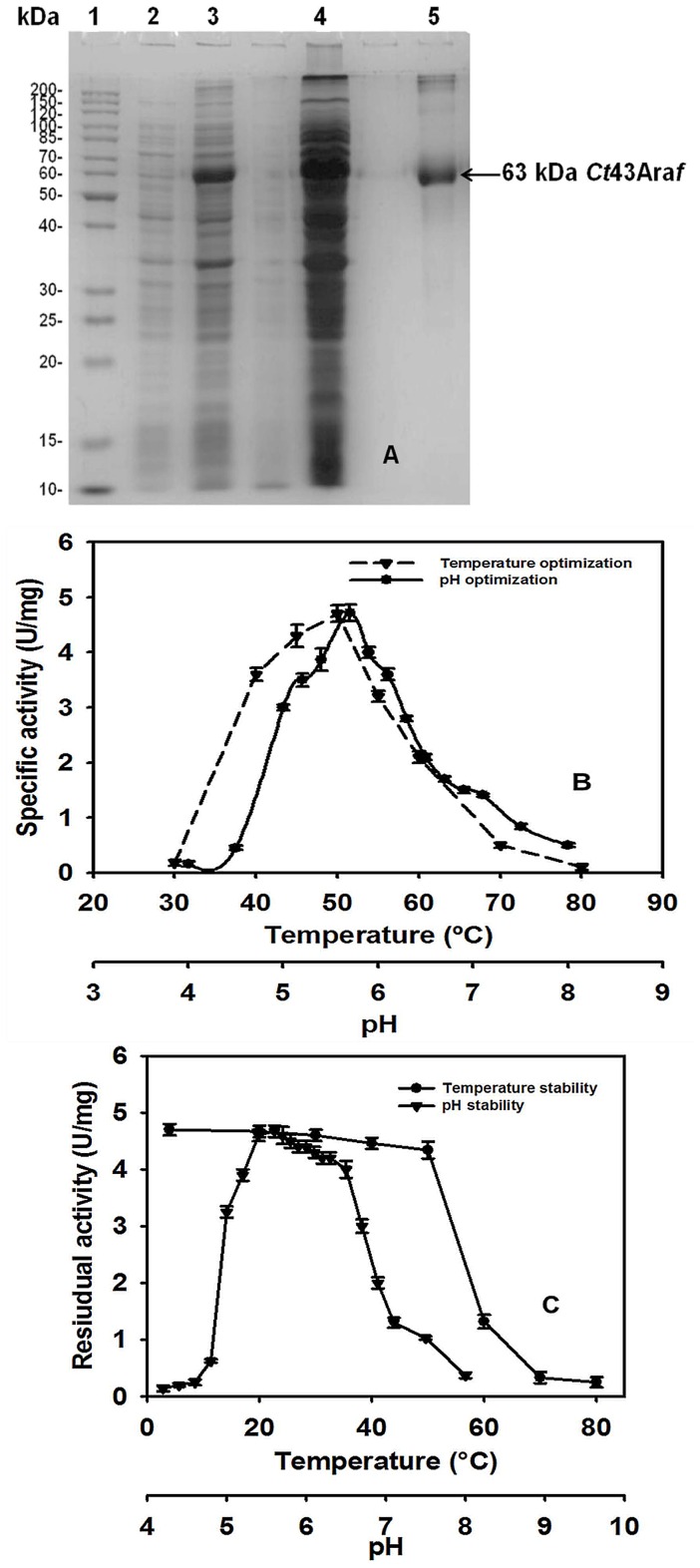
A) SDS-PAGE (13%) showing over-expression and purification of *Ct*43Ara*f*. Lane 1: Page Ruler protein marker, Lane 2: uninduced *Ct*43Ara*f* cells, Lane 3: Induced *Ct*43Ara*f* cells, Lane 4: Cell free extract, Lanes 5: Purified *Ct*43Ara*f* (63 kDa approx.), **B**) Effect of pH and temperature on *Ct*43Ara*f* activity, where (•) represents pH profile and (▾) represents temperature profile, **C**) pH and thermal stability analysis of *Ct*43Ara*f,* where (▾) represents pH stability and (•) represents thermal stability profile.

After confirming the positive clones by restriction enzyme digestion, *E. coli* BL-21(DE3) cells were transformed with recombinant plasmid of *Ct*43Ara*f*. *Ct*GH43 was transformed in *E. coli* BL-21 (DE3) pLysS cells [21]. The expression of recombinant proteins were analysed by SDS-PAGE. The His_6_-tagged clostridial recombinant proteins were purified by immobilized metal ion affinity chromatography (IMAC) from the cell free extracts. The SDS-PAGE analysis of purified recombinant protein *Ct*43Ara*f* is displayed in Fig. 2A. The recombinant protein *Ct*43Ara*f* displayed molecular size of 63 kDa, whereas, *Ct*GH43 showed a size of 34 kDa as reported previously [21]. The above recombinant proteins were expressed as soluble proteins. The ability of *Ct*43Ara*f* and *Ct*GH43 to hydrolyze the arabinose-containing polysaccharides was explored and compared.

### Effect of pH and Temperature on *Ct*43Ara*f* and *Ct*GH43

*Ct*43Ara*f* showed maximum enzyme activity (4.7 U/mg) at a pH of 5.7 and was observed to be stable in the pH range of 5.0–6.5 (Fig. 2B). The maximum enzyme activity of *Ct*43Ara*f* was observed at 50°C and the enzyme was found to be stable up to 50°C (Fig. 2C) which is consistent with the pH and temperature profiles of previously reports of recombinant proteins from *Clostridium thermocellum* by Taylor *et al*. 2005 [33] and Lee *et al*. 2013 [34].

### Substrate Specificity and Kinetic Parameters of Recombinant *Ct*43Ara*f* and *Ct*GH43

#### Substrate specificity and kinetic parameters with natural substrates

The assays for natural substrates were carried out using the pH, buffers and other conditions as described in methods section. The activities of *Ct*43Ara*f* and its truncated derivative *Ct*GH43 with various polysaccharides are reported in Table 1. The maximum specific activity (U mg^−1^) of *Ct*43Ara*f* and *Ct*GH43 were found to be 4.7 and 5.0, respectively, with rye arabinoxylan which was followed by the decreasing order of activities against wheat arabinoxylan, oat spelt xylan, beechwood xylan and birchwood xylan as illustrated in Table 1. Both the catalytic enzymes showed low activity (less than 1.0 Umg^−1^) with arabinogalactan and rhamnogalactouronan (*α*-L-arabinopyranosyl side chain containing polysaccharides) [Table 1]. *Ct*43Ara*f* and *Ct*GH43 did not show any considerable increase in activity with 1,5-α-L-arabinobiose and 1,5-α-L-arabinotetraose (the enzymatically hydrolyzed de branched sugar beet arabinan) as compared to complex sugar beet arabinan which indicated that the above enzymes lack specificity for 1,5-α-L-arabinosyl linkages [Table 1]. The above results also indicated that both *Ct*43Ara*f* and *Ct*GH43 do not possess α-L-arabinanase type of activity. The kinetic properties and catalytic efficiency of both the enzymes were determined with the natural substrates [Table 2]. *Ct*43Ara*f* and *Ct*GH43 showed highest turnover number (k_cat_) of 280 min^−1^ and 298 min^−1^, respectively, and also highest catalytic efficiency (k_cat_/*K*_m_, min^−1^ mg^−1^ ml) of 3.4×10^3^ and 3.6×10^3^ with rye arabinoxylan. Both the enzymes were able to act on insoluble wheat arabinoxylan showing catalytic efficiencies (k_cat_/*K*_m_) of 7.1×10^2^ min^−1^mg^−1^ml and 6.1×10^2 ^min^−1^mg^−1^ml for *Ct*43Ara*f* and *Ct*GH43, respectively. The catalytic efficiencies (k_cat_/*K*_m_) observed with other soluble substrates like beechwood and birchwood xylans were found to be comparatively less.

**Table 1:** Substrate specificity of *Ct*43Ara*f* and *Ct*GH43 from *C. thermocellum.*

Substrates (polysaccharides)	Specific Activity of^a^ *Ct*43Ara*f* (U/mg)	Specific Activity of^b^ *Ct*GH43 (U/mg)
Arabinoxylan, (Rye)	4.70±0.07	5.00±0.08
Arabinoxylan (wheat, soluble)	2.50±0.03	2.70±0.03
Xylan (Oat spelt)	1.70±0.08	1.80±0.07
Arabinoxylan (wheat, insoluble)	1.20±0.10	1.10±0.10
Xylan (Beechwood)	1.00±0.04	0.90±0.04
Xylan (Birchwood)	0.70±0.03	0.80±0.04
Arabinogalactan	0.25±0.05	0.32±0.05
Arabinan (sugar beet)	0.22±0.04	0.23±0.04
Carboxy methyl cellulose	ND	ND
Carboxy ethylcellulose	ND	ND
**Substrates (oligosaccharides)**		
*1,5-α-L-arabinobiose	0.25±0.04	0.24±0.04
**1,5-α-L-arabinotetraose	0.22±0.04	0.20±0.04

*All the assays were performed at 50°C using 20 mM sodium phosphate (pH 5.7) buffer for ^a^Ct43Araf and 20 mM sodium acetate (pH 5.4) buffer for ^b^CtGH43. The assays were performed in triplicates. The incubation time and other conditions for reducing sugar estimation were as same as described in the Materials and Methods section.*

* /**It was prepared by Megazyme (Ireland) via controlled enzymatic hydrolysis of de branched sugar beet arabinan as described in the manufacturer’s instructions.

ND = *No activity detected.*

**Table 2:** Kinetic properties and catalytic efficiencies of *Ct*43Ara*f* and *Ct*GH43 from *C. thermocellum.*

Substrates	*K*_m_(mg ml^−1^)	*k*_cat_(min^−1^)	*k*_cat_/*K*_m_(min^−1^mg^−1^ml^−1^)
**^a^** ***Ct*** **43Ara** ***f***			
**Natural polysaccharides**			
Rye arabinoxylan	0.082±0.005	280.0. ±4	3.4×10^3^
Wheat arabinoxylan (soluble)	0.072±0.003	190.0±2	2.6×10^3^
Wheat arabinoxylan(insoluble)	0.09±0.015	63.0±12	7.1×10^2^
Oat spelt xylan	0.085±0.005	65.0±3.0	7.7×10^2^
Birchwood xylan	0.95±0.004	27.0±0.9	2.8×10^1^
Beechwood xylan	0.7±0.004	28.0±0.5	4.0×10^1^
**^c^Synthetic ** ***p*** **NP-glycosides**			
*p*NP-*α*-L-arabinofuranoside	0.05±0.002	283.0±2.0	5.6×10^3^
*p*NP-*α*-L-arabinopyranoside	0.093±0.003	210.0±4.7	2.2×10^3^
**^b^** ***Ct*** **GH43**			
**Natural polysaccharides**			
Rye arabinoxylan	0.08±0.002	298.0±8.0	3.6×10^3^
Wheat arabinoxylan (soluble)	0.078±0.005	209.0±2.0	2.7×10^3^
Wheat arabinoxylan(insoluble)	0.1±0.01	61.0±18	6.1×10^2^
Oat spelt xylan	0.08±0.002	67.0±2.0	8.3×10^2^
Birchwood xylan	0.9±0.002	29.0±1.0	3.2×10^1^
Beechwood xylan	0.8±0.003	26.0±2.0	3.3×10^1^
**^c^Synthetic ** ***p*** **NP-glycosides**			
*p*NP-*α*-L-arabinofuranoside	0.04±0.004	287.0±1.0	7.1×10^3^
*p*NP-*α*-L-arabinopyranoside	0.097±0.004	212.0±2.0	2.2×10^3^

*The assays with natural substrates were carried out with 20 mM sodium phosphate buffer (pH 5.7) for **^a^**Ct43Araf and sodium acetate buffer (pH 5.4) for **^b^**CtGH43 at 50°C. The assays were performed in triplicates. The incubation time and other conditions for reducing sugar estimation were as same as described in the Materials and Methods section. **^c^**The assays with synthetic pNP-glycosides were carried out in 20 mM sodium phosphate buffer pH 5.7.*

#### Substrate specificity and kinetic parameters with synthetic substrates

The catalytic efficiencies (k_cat_/*K*_m_) of *Ct*43Ara*f* and *Ct*GH43 with *p*NP-*α*-L-arabinofuranoside (*p*NPA*f*) were found to be 5.6×10^3^ min^−1^ mg^−1^ ml and 7.1×10^3^ min^−1^ mg^−1^ ml, respectively, while with *p*NP-*α*-L-arabinopyranoside (*p*NPA*p*), the k_cat_/*K*_m_ value for both the modules was 2.2×10^3^ min^−1^ mg^−1^ ml [Table 2]. Therefore, we can say that the *Ct*43Ara*f* and *Ct*GH43 were able to release *p*-nitrophenol from *p*NP-*α*-L-arabinofuranoside (*p*NPA*f*) as well as from *p*NP-*α*-L-arabinopyranoside (*p*NPA*p*), but the catalytic efficiencies of both modules were approximately, 3-fold higher with *p*NPA*f* as compared to *p*NPA*p*. Based on the catalytic efficiencies observed for *Ct*43Ara*f* and *Ct*GH43 with natural as well as synthetic substrates, it is evident that both these proteins are predominantly *α*-L-arabinofuranosidase.

### Effects of Metal Ions and Chemical Agents on *Ct*43Araf and *Ct*GH43 Activity

The enzyme activities of *Ct*43Ara*f* and *Ct*GH43 increased by more than 2-fold with Mg^2+^ and Ca^2+^ salts at low concentrations (5–10 mM) [Table 3]. A slight increase in activity of *Ct*43Araf and *Ct*GH43 were also observed with Ni^2+^ salts (15% and 47%), Mn^2+^ (20% and 21%) and Zn^2+^ (8% and 24%) salts [Table 3]. The enzyme activity of *Ct*43Ara*f* and *Ct*GH43 were adversely affected at higher concentrations (20 mM) of Co^2+^ (75% in both enzymes), Hg^2+^ (80% and 70%), Cu^2+^ (70% in both enzymes) and Ag^+^ (80% and 75%), respectively [Table 3]. The enzyme activity of both *Ct*43Ara*f* and *Ct*GH43 decreased by more than 90% in presence of EDTA (10 mM) or 10 mM EGTA [Table 3]. The decrease in activity in presence of EGTA indicated that Ca^2+^ ions may be essential for enzyme activity as EGTA specifically binds and chelates calcium ions in 1∶1 molar ratio [35]. The catalytic activity was noticeably increased in the presence of Ca^2+^ and Mg^2+^salts elucidating the fact that these metal cations may be needed as co-factors while the heavy metals especially Co^2+^, Hg^2+^, Cu^2+^ and Ag^+^ caused decrease in enzyme activity as shown for recombinant cellulases [36], [37].

**Table 3:** Maximum effect on enzyme activity of *Ct*43Ara*f* and *Ct*GH43 from *Clostridium thermocellum* at maximum concentration of metal ions and chelating agents.

Metal ion/Reagent	Concentration of additives (mM)	Relative activity (%)	
		*Ct*43Ara*f*	*Ct*GH43
Control	–	100	100
Na^+^	50.0	100	100
Ca^2+^	8.0	216	217
Mg^2+^	6.0	211	207
Ni^2+^	4.0	115	147
Zn^2+^	2.0	124	108
Mn^2+^	4.0	120	121
Fe^3+^	20.0	50	50
Al^3+^	20.0	50	50
Cu^2+^	10.0	30	30
Co^2+^	20.0	25	25
Hg^2+^	20.0	20	30
Ag^+^	20.0	20	25
EDTA	20.0	05	05
EGTA	20.0	05	05

*No additives were added in control and the activity was taken as 100%.*

### TLC Analysis of Enzyme Reaction Products

**Figure 3:**
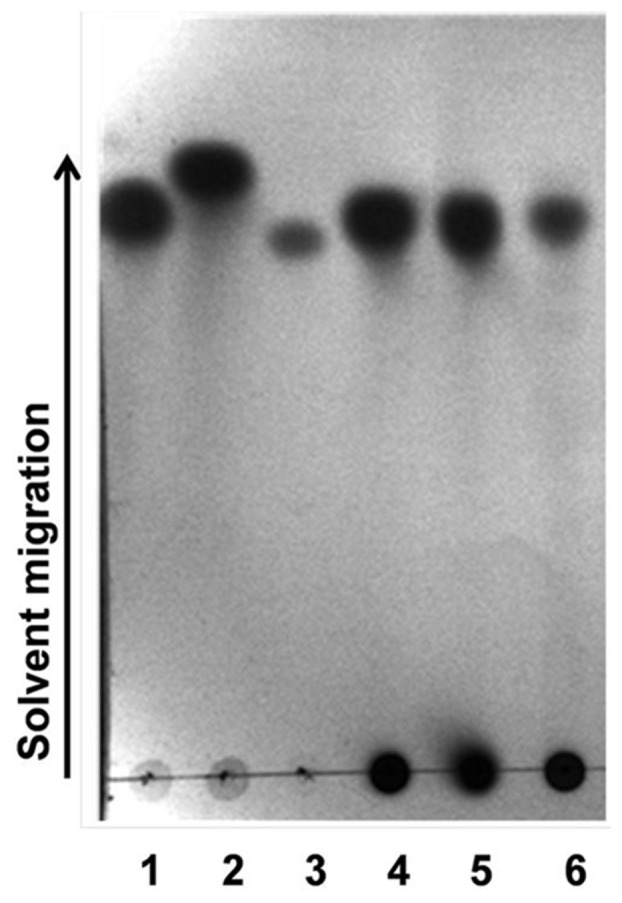
Thin layer chromatography analysis of reaction products of *Ct*43Ara*f*. Dark spots on TLC plate show the standards L-arabinose, D-xylose and cellobiose (spots 1, 2 and 3, respectively) while spots 4, 5 and 6 represent hydrolyzed products from rye arabinoxylan, wheat arabinoxylan and oat spelt xylan, respectively, showing that only L-arabinose is released as the breakdown product.

The TLC analysis of the enzyme reaction products (sugars) indicated that *Ct*43Ara*f* releases only arabinose from rye arabinoxylan, wheat arabinoxylan and oat spelt xylan [Fig. 3]. The similar results were obtained with beechwood xylan and insoluble wheat arabinoxylan (data not shown). The above result and the results obtained from the assays with *p*NP-glycosides indicated that *Ct*43Ara*f* could be α-L-arabinofuranosidase since the above mentioned polysaccharides contain α-L-arabinofuranosyl residues. The relative migration of *Ct*43Ara*f* hydrolyzed sugars with commercially available standards clearly indicated that L-arabinose could possibly be the major sugar released as no spots for xylose was observed on the TLC plate [Fig. 3]. This was in agreement with the previous reports on *α*-L-arabinofuranosidases [38].

### HPAEC Analysis of Enzyme Reaction Products

**Figure 4:**
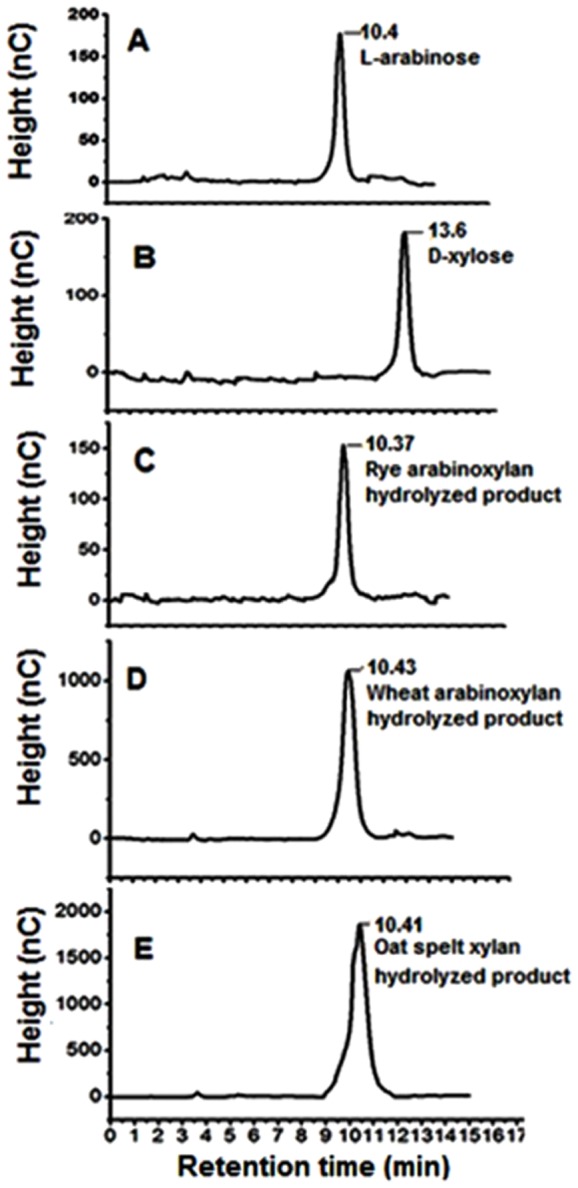
HPAEC analysis of *Ct*43Ara*f* reaction mixture showing released sugars. **A**) L-arabinose, **B**) D-xylose, **C**) rye arabinoxylan, **D**) wheat arabinoxylan and **E**) oat spelt xylan. The reaction was carried out at pH 5.7, 50°C for 30 min.

The reaction products of *Ct*43Ara*f* with rye arabinoxyalan, wheat arabinoxylan (soluble) and oat spelt xylan analyzed by HPAEC showed only arabinose as the released sugar [Fig. 4]. L-Arabinose and D-xylose used as standards for the HPEAC analysis of enzyme-substrate reaction products showed peaks at 10.4 min and at 13.6 min, respectively [Fig. 4A and 4B]. The chromatograms of hydrolysis products (rye arabinoxylan, wheat arabinoxylan and oat spelt xylan) by both *Ct*43Ara*f* and *Ct*GH43 showed only the peak corresponding to L-arabinose at 10.4 min and no peak for xylose was observed [Fig. 4C, 4D and 4E]. Based on HPAEC and TLC analysis and the results obtained with *p*-NP-glycoside assays we can conclude that both *Ct*43Ara*f* and *Ct*GH43 exhibit *α*-L-arabinofuranosidase activity as also reported earlier [38]. The HPAEC analysis of the hydrolyzed products of *α*-L-arabinofuranosidase *Ct*43Ara*f* from *C. thermocellum* supported the observation that both the modules released L-arabinose from arabinoxylans.

### Protein Melting-curve Analysis *Ct*43Ara*f* and *Ct*GH43

**Figure 5:**
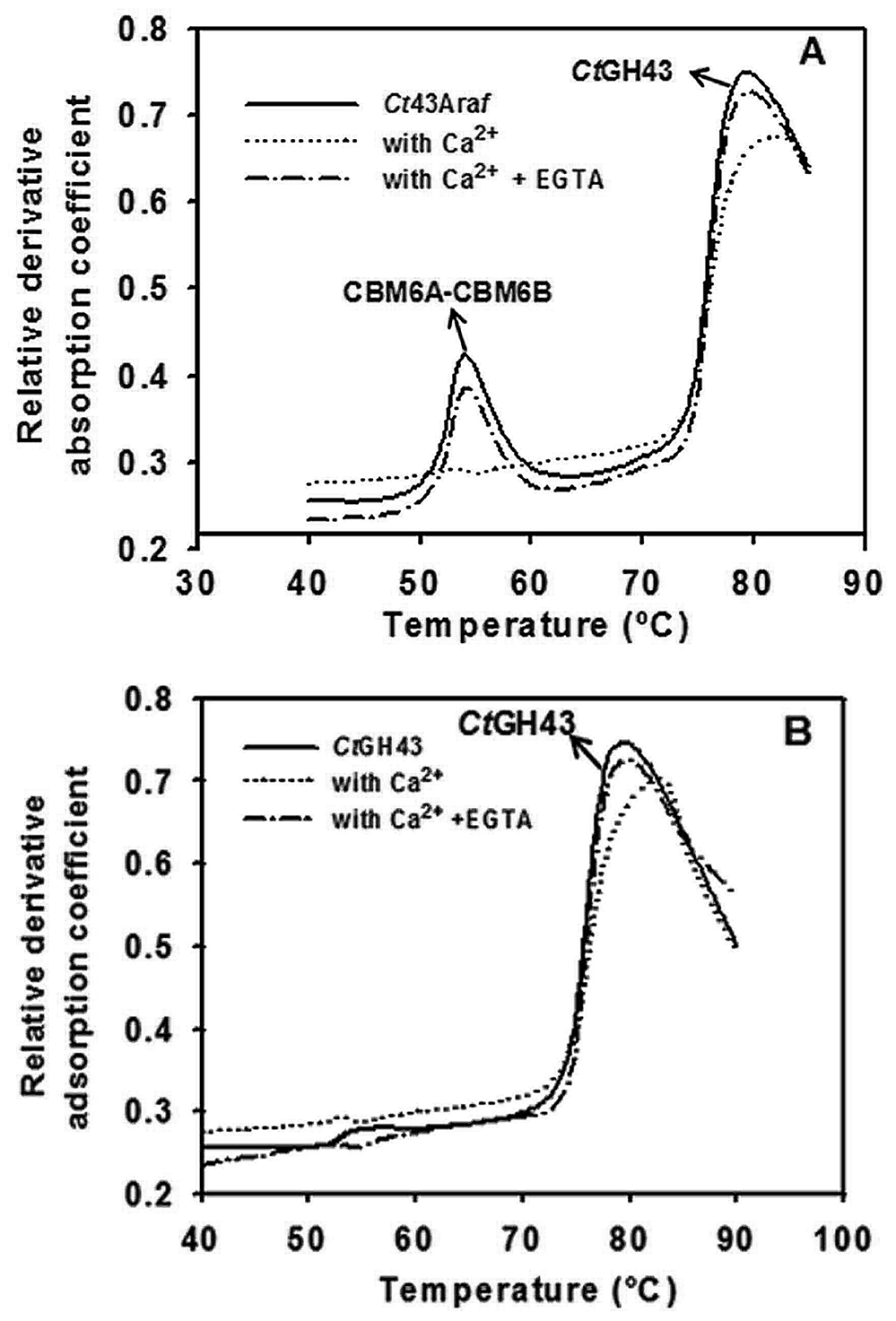
Protein-melting analysis displaying normal melting curve (–), melting curve in presence of 10 mM Ca^2+^ ions (–), and melting curve in presence of 10 mM Ca^2+^ ions and 10 mM EGTA (–•–), A) Melting-profile of *Ct*43Ara*f* and B) melting profile of truncated derivative, *Ct*GH43.

The recombinant protein *Ct*43Ara*f* showed two separate melting peaks at 53°C and 78°C [Fig. 5A], whereas, *Ct*GH43 displayed a single melting peak at around 78°C [Fig. 5B]. This suggested that the peak at 53°C is associated with CBMs (CBM6A-CBM6B) and the peak at 78°C was due to *Ct*GH43. This type of melting curve indicated that *Ct*GH43 and non-catalytic CBMs (CBM6A-CBM6B) are melting independently [Fig. 5A]. The presence of Ca^2+^ ions (10 mM) caused significant changes in *Ct*43Ara*f* as well as in *Ct*GH43 protein-melting profiles. The peak for *Ct*GH43 shifted towards higher temperature i.e. 83°C from 78°C but the peak for corresponding to CBMs (CBM6A-CBM6B) of *Ct*43Ara*f* was masked in presence of Ca^2+^ ions [Fig. 5 A & 5B]. When EGTA salt was added (10 mM) to the enzyme-substrate reaction mixture containing Ca^2+^ (10 mM), there was shifting back of the melting peaks to the original temperature of 78°C of catalytic *Ct*GH43 as evident from Fig. 5 A & B (small dotted lines).

### Structural Analysis of *Ct*GH43 by Circular Dichroism

**Figure 6:**
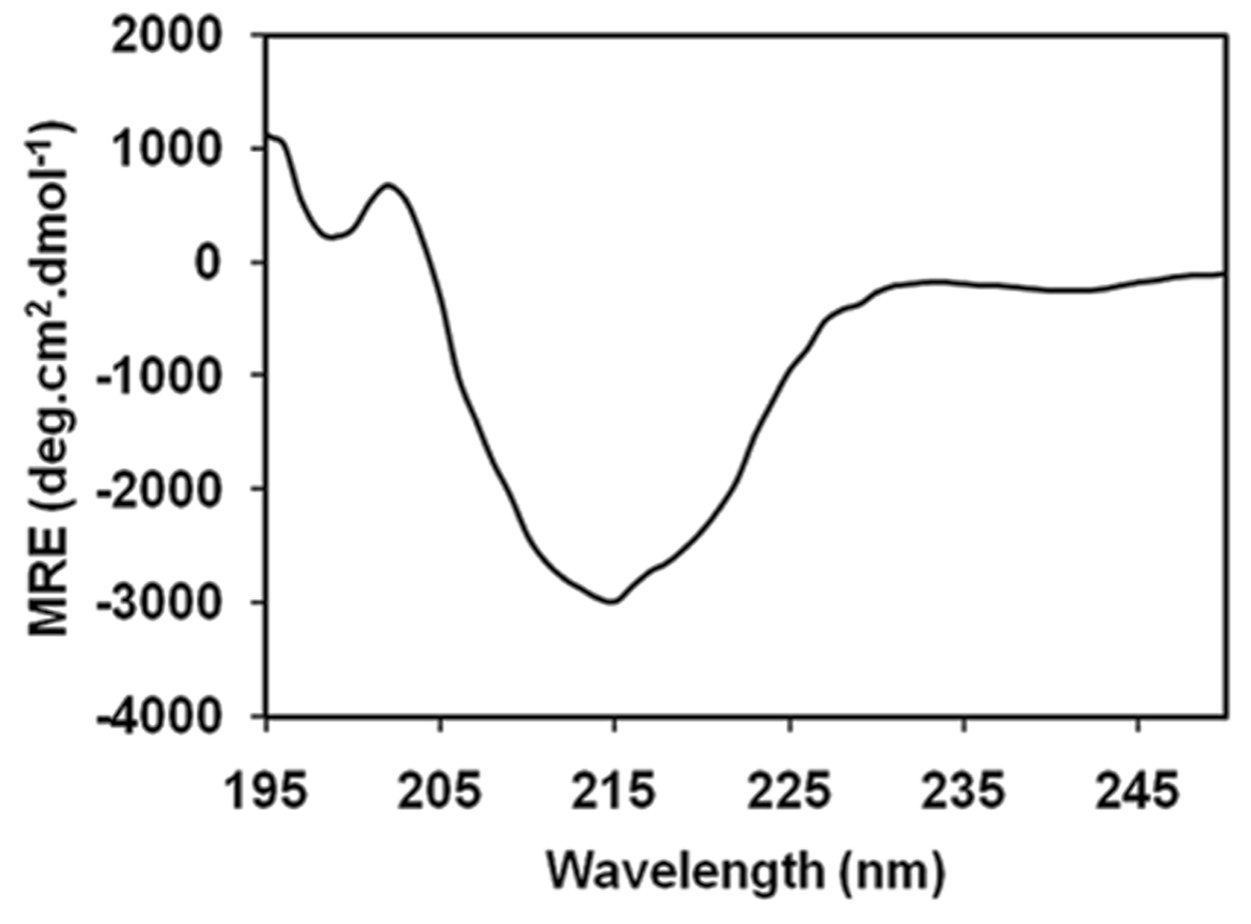
Far-UV CD spectra of truncated *Ct*GH43 (15 µM) from *Clostridium thermocellum* in 20 mM sodium phosphate buffer, pH 7.0.

The analysis of CD spectra of *Ct*GH43 for detecting the secondary structural elements was based on the previous reports of CD spectra analysis of proteins by Kelly *et al.* [31] which showed that it mostly contained β-sheets and random coils [Fig. 6]. The CD spectra *Ct*GH43 was analysed using K2d as described by Perez-Iratxeta and Andrade-Navarro, [32] revealed that it contains 48% β-sheets, 49% random coils and only 3% α-helices [Table 4].

**Table 4:** The percentage of secondary structure contents of *Ct*GH43 protein as estimated from far-UV CD spectra.

Secondary structurecontents of *Ct*GH43	Percentage (%)by CD analysis	Percentage (%)by PSIPRED VIEW*
α-helix	03	00
β-sheet	48	51
Random Coil	49	49

* *Secondary structure prediction using PSIPRED VIEW software.*

## Discussion

In recent past, few family 43 glycoside hydrolases have been reported in the CAZy database (www.cazy.org) from *C. thermocellum* (galactanase), *B. thetaiotaomicron (α*-1,2-arabinofuranosidase), *C. japonicas* (*α*-1,5-exoarabinanase) *B. adolescentis* (only other known arabinofuranosidase with ability to hydrolyze doubly substituted xylans) [6], [9], [13], [14]. *Ct*GH43 unlike other member of the family 43 glycoside hydrolase showed significant homology with CBM6s from different bacterial sources [39]. The sequence and phylogenetic tree analysis of *Ct*GH43 has been reported by Ahmed *et al*., 2012 [39]. *Ct*43Ara*f* and *Ct*GH43 showed maximum activity against rye arabinoxylan; however, significant activities were also observed with wheat arabinoxylan, oat spelt xylan, birchwood xylan and beech wood xylan. It has been previously reported that the rye arabinoxylans have nearly 50% of the xylose residues substituted at the terminal by L-arabinose at O-3 and around 2% at both O-2 and O-3. This suggested that *Ct*43Ara*f* displays *α*-1,3-arabinofuranosidase type of activity in exo-acting manner similar to the previous report by Bengtsson *et al.* [4]. *Ct*43Ara*f* and *Ct*GH43 also showed lesser but considerable activity with water soluble wheat arabinoxylan, which are rich in 2-mono and di-substituted xyloses and low in 3-mono and di-substituted xylose [4], [6], [9], indicating that they also act at O-2 substituted xylose. Further, *Ct*43Ara*f* and *Ct*GH43 displayed noticeable activities with beechwood and birchwood xylans comprising of O-2 and O-3 substituted xylose. The above observations and previous report by Bourgois *et al.* [12] indicated that both the recombinant enzymes have the ability to catalyze the hydrolysis of terminal non-reducing *α*-L-1,2- as well as *α*-L-1,3- arabinosyl residues in exo-acting manner. The activity with oat spelt xylan can be attributed to the presence of arabinose (10%, w/v). The low activity with arabinogalactan and rhamnogalactouronan was mainly due to the fact that similar to β-1,4-xylopyranose, the the β-1,4-galactans too, are poorly substituted with α-L-arabinopyranose side-chains as reported by Øbro *et al.* [8]. Therefore, we can conclude that enzymes (*Ct*43Ara*f* and *Ct*GH43) acted mainly on the glycosidic linkage of α-arabinfuranosyl substituted to the main chain β-1,4-xylose occurring predominantly in the arabinoxylans. Both *Ct*43Ara*f* and *Ct*GH43 were able to act on and degrade synthetic substrates *p*NP-α-L*-*arabinofuranoside as well as *p*NP-*α*-L-arabinopyranoside. Both *Ct*43Ara*f* and *Ct*GH43 showed high catalytic efficiencies against *p*NPA*f* and *p*NPA*p,* elucidating their bi-functional nature. But close inspection of catalytic efficiency data revealed that *Ct*43Ara*f* is primarily *α*-L-arabinofuranosidase as the *k*_cat_/*K*_m_ was 3-fold higher in case of *p*NPA*f* as compared to *p*NPA*p*.

The enzyme activity of *Ct*43Ara*f* and *Ct*GH43 increased significantly by more than two-fold in presence of Ca^2+^ and Mg^2+^ salts, implying that these ions are needed as cofactors. However, the enzyme activity was unaffected by lower concentrations of Cu^2+^, Co^2+^, Hg^2^ or Ag^+^ ions but it decreased at higher concentrations. The enzyme activity of *Ct*43Ara*f* and *Ct*GH43 decreased sharply in the presence of EGTA. This implied that Ca^2+^ ions might be involved in the catalysis and imparting stability to the structures of *Ct*43Ara*f* and *Ct*GH43. A few recombinant family 43 glycoside hydrolases have been reported in the past by Sanctis *et al*., [40], Morais *et al*., [41] and Jordan *et al*., [42] which showed enhanced enzyme activity in the presence of Ca^2+^ ions.

The TLC and HPAEC analyses indicated that both *Ct*43Ara*f* and *Ct*GH43 released L-arabinosyl side chain sugars from arabinoxylans. The TLC analysis of the hydrolysis products of rye arabinoxylan, wheat arabinoxylan and oat spelt xylan with *Ct*43Ara*f* indicated that L-arabinose is the main sugar that was released as a result of enzyme substrate reaction. HPAEC analysis of the hydrolysis products of *Ct*43Ara*f* with rye arabinoxylan, wheat arabinoxylan and oat spelt xylan further corroborated the above observation that L-arabinose is the only monosaccharide released after the hydrolysis. However, close inspection of the TLC and HPAEC results combined with the results obtained with synthetic *p*NP-glycosides confirmed that both *Ct*43Ara*f* and *Ct*GH43 are predominantly α-L-arabinofuranosidase since both showed higher catalytic efficiency with *p*NPA*f*.

Protein-melting curves of *Ct*43Ara*f* and *Ct*GH43 showed that the *Ct*GH43 and CBMs (CBM6A-CBM6B) melt independently of each other. The protein-melting peaks of *Ct*GH43 and CBMs shifted to higher temperature in the presence of Ca^2+^ ions. However, on addition of equimolar concentration of EGTA and Ca^2+^ ions to the solutions of *Ct*43Ara*f* and *Ct*GH43, the melting temperature peaks were shifted back to the original positions. The presence of Ca^2+^ ions stabilized both the CBMs and *Ct*GH43 module and prevented unfolding or denaturation. This enhanced stability of the CBM modules is observed through the absence of a 53°C denaturation event.. Similar observations were also reported with a β-1,3-glucanase and associated CBMX module [30]. The independent melting of protein modules was deduced by comparing with the previously reported melting profile of protein modules [30], [43]. The shifting back of melting peaks in the presence of EGTA can be explained by the highly specific chelation of calcium ions by EGTA, making them unavailable to stabilize the enzyme CBM modules.

The CD analysis confirmed the fact that β-sheets and random coils were the main secondary structural elements present in the recombinant protein *Ct*GH43. Only 3% α-helices were present in the *Ct*GH43 structure. The results of CD analysis of *Ct*GH43 were in agreement with the secondary structure prediction of the same protein using PSIPRED VIEW software [44] with slight difference in number of α-helices [Table 4]. However, it is important to consider that the presence of aromatic residues can significantly affect the far-UV CD spectrum of peptides as reported by Pace and Scholtz [45] and Fujiwara *et al*., [46]. Such interactions sometimes could produce a significant increase in the helical population.

### Conclusions

The *Ct*43Ara*f* and its truncated derivative *Ct*GH43 possess α-L-arabinofuranosidase type of activity as analyzed by *p*NP-glycoside based assays, TLC and HPAEC analysis. The presence of CBMs did not affect the α-L-arabinofuranosidase activity of *Ct*43Ara*f* and *Ct*GH43. The enzyme activity of both *Ct*43Ara*f* and *Ct*GH43 was significantly enhanced in the presence of Ca^2+^ and Mg^2+^ salts. The *Ct*43Ara*f* and *Ct*GH43 showed ability to degrade both *p*-nitrophenol-*α*-L-arabinofuranoside and *p*-nitrophenol-*α*-L-arabinopyranoside. The presence of Ca^2+^ ions imparted thermal stability to both the enzymes. The circular dichroism analysis of *Ct*GH43 showed that it is mostly composed of β-sheets and random coils.

## References

[1] Harris PJ, Stone BA (2009) Chemistry and molecular organization of plant cell walls. In: Biomass recalcitrance: deconstructing the plant cell wall for bioenergy (ed. Himmel ME), Blackwell Publishing Ltd: Oxford, UK. 61–93.

[2] Saha BC (2003) Hemicellulose bioconversion J Ind Microbiol Biotechnol. 30: 279–291. 10.1007/s10295-003-0049-x 12698321

[3] Ordaz-Ortiz JJ, Saulnier L (2005) Structural variability of arabinoxylans from wheat flour, comparison of water-extractable and xylanase-extractable arabinoxylans J Cereal Sci. 42: 119–125.

[4] Bengtsson S, Aman P, Andersson RE (1992) Structural studies on water-soluble arabinoxylans in rye grain using enzymatic hydrolysis Carbohyd Polym. 17: 277–284.

[5] Gruppen H, Hamer RJ, Voragen AGJ (1992) Water-unextractable cell-wall material from wheat-flour, fractionation of alkali-extracted polymers and comparison with water-extractable arabinoxylans J Cereal Sci. 16: 53–67.

[6] Numan MT, Bhosle MB (2006) α-L-Arabinofuranosidases: the potentials applications in biotechnology J Ind Microbiol Biotechnol. 33: 247–260. 10.1007/s10295-005-0072-1 16385399

[7] Morris PC, Bryce JH (2000) Cereal Biotechnology, CRC Press, Woodhead Publishing Limited, Cambridge, England.

[8] Øbro J, Harholt J, Scheller HV, Orfila C (2004) Rhamnogalacturonan I in *Solanum* tuberosum tubers contains complex arabinogalactan structures Phytochemistry. 65: 1429–1438. 10.1016/j.phytochem.2004.05.002 15231417

[9] Saha BC (2000) Alpha-L-arabinofuranosidases: biochemistry, molecular biology and application in biotechnology Biotechnol Adv. 18: 403–423. 10.1016/s0734-9750(00)00044-6 14538102

[10] Pason P, Kosugi A, Waeonukul R, Tachaapaikoon C, Ratanakhanokchai K, et al (2010) Purification and characterization of a multienzyme complex produced by *Paenibacillus curdlanolyticus* B-6 Appl Microbiol Biotechnol. 85: 573–580. 10.1007/s00253-009-2117-2 19597812

[11] Zhou J, Bao L, Chang L, Zhou Y, Lu H (2012) Biochemical and kinetic characterization of GH43 β-d-xylosidase/α-L-arabinofuranosidase and GH30 α-l-arabinofuranosidase/β-d-xylosidase from rumen metagenome J Ind Microbiol Biotechnol. 39: 143–152. 10.1007/s10295-011-1009-5 21720773

[12] Bourgois TM, Van Craeyveld V, Van Campenhout S, Courtin CM, Delcour JA, et al (2007) Recombinant expression and characterization of XynD from *Bacillus subtilis* subsp subtilis ATCC 6051: a GH43 arabinoxylan arabinofuranohydrolase Appl Microbiol Biotechnol. 75: 1309–1317. 10.1007/s00253-007-0956-2 17426966

[13] Cartmell A, McKee LS, Peña MJ, Larsbrink J, Brumer H, et al (2011) The structure and function of an arabinan-specific α-1,2-Arabinofuranosidase identified from screening the activities of bacterial GH43 glycoside hydrolases J Biol Chem. 286: 15483–15495. 10.1074/jbc.M110.215962 PMC3083193 21339299

[14] Sørensen HR, Jørgensen CT, Hansen CH, Jørgensen CI, Pedersen S, et al (2006) A novel GH43 alpha-L-arabinofuranosidase from *Humicola insolens*: mode of action and synergy with GH51 alpha-L-arabinofuranosidases on wheat arabinoxylan Appl Microbiol Biotechnol. 73: 850–861. 10.1007/s00253-006-0543-y 16944135

[15] Adelsberger H, Hertel C, Glawischnig E, Zverlov VV, Schwarz WH (2004) Enzyme system of *Clostridium stercorarium* for hydrolysis of arabinoxylan: reconstitution of the *in vivo* system from recombinant enzymes Microbiology. 150: 2257–2266. 10.1099/mic.0.27066-0 15256568

[16] Taylor EJ, Smith NL, Turkenburg JP, D’Souza S, Gilbert HJ, et al (2006) Structural insight into the ligand specificity of a thermostable family 51 arabinofuranosidase, Araf51, from *Clostridium thermocellum* Biochem J. 395: 31–37. 10.1042/BJ20051780 PMC1409695 16336192

[17] Guais O, Tourrasse O, Dourdoigne M, Parrou JL, Francois JM (2010) Characterization of the family GH54 α-l-arabinofuranosidases in *Penicillium funiculosum*, including a novel protein bearing a cellulose-binding domain Appl Microbiol Biotechnol. 87: 1007–1021. 10.1007/s00253-010-2532-4 20333513

[18] Sakamoto T, Ogura A, Inui M, Tokuda S, Hosokawa S, et al (2011) Identification of a GH62 α-l-arabinofuranosidase specific for arabinoxylan produced by *Penicillium chrysogenum* Appl Microbiol Biotechnol. 90: 137–146. 10.1007/s00253-010-2988-2 21181156

[19] Hashimoto K, Yoshida M, Hasumi K (2011) Isolation and characterization of *Cc*Abf62A, a GH62 α-L-arabinofuranosidase, from the basidiomycete *Coprinopsis cinerea* Biosci Biotechnol Biochem. 75: 342–345. 10.1271/bbb.100434 21307589

[20] Aristidou A, Penttilä M (2000) Metabolic engineering applications to renewable resource utilization Curr Opin Biotechnol. 11: 187–198. 10.1016/s0958-1669(00)00085-9 10753763

[21] Das SP, Ravindran R, Ahmed S, Das D, Goyal D, et al (2012) Bioethanol Production involving recombinant *C thermocellum* hydrolytic hemicellulase and fermentative microbes Appl Biochem Biotechnol. 127: 1475–1488. 10.1007/s12010-012-9618-7 22383050

[22] Osaki S, Kimura T, Sugimoto T, Hizukuri S, Iritani N (2001) L-Arabinose feeding prevents increases due to dietary sucrose in lipogenic enzymes and triacylglycerol levels in rats J Nutr. 131: 796–799. 10.1093/jn/131.3.796 11238761

[23] Boraston AB, Bolam DN, Gilbert HJ, Davies GJ (2004) Carbohydrate-binding modules: fine-tuning polysaccharide recognition Biochem J. 382: 769–781. 10.1042/BJ20040892 PMC1133952 15214846

[24] Abbott DW, Ficko-Blean E, van Bueren AL, Rogowski A, Cartmell A, et al (2009) Analysis of the structural and functional diversity of plant cell wall specific family 6 carbohydrate binding modules Biochemistry. 48: 10395–10404. 10.1021/bi9013424 19788273

[25] Ichinose H, Yoshida M, Fujimoto Z, Kaneko S (2008) Characterization of a modular enzyme of exo-1,5-alpha-L-arabinofuranosidase and arabinan binding module from *Streptomyces avermitilis* NBRC14893 Appl Microbiol Biotechnol. 80: 399–408. 10.1007/s00253-008-1551-x PMC2518083 18665359

[26] Laemmli UK (1970) Cleavage of structural proteins during the assembly of the head of bacteriophage T4 Nature. 227: 680–685. 10.1038/227680a0 5432063

[27] Nelson N (1944) A photometric adaptation of the Somogyi method for the determination of glucose J Biol Chem. 153: 375–380.

[28] Somogyi M (1945) A new reagent for the determination of sugars J Biol Chem. 160: 61–68.

[29] Cote GL, Leathers TD (2005) A method for surveying and classifying *Leuconostocs* pp Glucansucrases according to strain-dependent acceptor product patterns J Ind Microbiol Biotechnol. 32: 53–60. 10.1007/s10295-004-0194-x 15714308

[30] Dvortsov IA, Lunina NA, Chekanovskaya LA, Schwarz WH, Zverlov VV, et al (2009) Carbohydrate-binding properties of a separately folding protein module from beta-1,3-glucanase Lic16A of *Clostridium thermocellum* Microbiology. 155: 2442–2449. 10.1099/mic.0.026930-0 19389758

[31] Kelly SM, Jess TJ, Price NC (2005) How to study proteins by circular dichroism Biochim Biophys Acta. 1751: 119–139. 10.1016/j.bbapap.2005.06.005 16027053

[32] Perez-Iratxeta C, Andrade-Navarro MA (2008) K2D2: estimation of protein secondary structure from circular dichroism spectra BMC Struct Biol 13. 8: 25. 10.1186/1472-6807-8-25 PMC2397409 18477405

[33] Taylor EJ, Goyal A, Guerreiro CI, Prates JA, Money VA, et al (2005) How family 26 glycoside hydrolases orchestrate catalysis on different polysaccharides: structure and activity of a *Clostridium thermocellum* lichenase, CtLic26A J Biol Chem. 280: 32761–32777. 10.1074/jbc.M506580200 15987675

[34] Lee CC, Braker JD, Grigorescu AA, Wagschal K, Jordan DB (2013) Divalent metal activation of a GH43 β-xylosidase Enzyme Microb Technol. 52: 84–90. 10.1016/j.enzmictec.2012.10.010 23273276

[35] Qin N, Olcese R, Bransby M, Lin T, Birnbaumer L (1999) Ca^2+^-induced inhibition of the cardiac Ca^2+^ channel depends on calmodulin Proc Natl Acad Sci USA. 96: 2435–2438. 10.1073/pnas.96.5.2435 PMC26802 10051660

[36] Bharali S, Prama RK, Majmder A, Fontes CMGA, Goyal A (2005) Molecular cloning and biochemical properties of family 5 glycoside hydrolase of bi-functional cellulase from *Clostridium thermocellum* Indian J Microbiol. 45: 317–321.

[37] Ahmed S, Bharali S, Purama RK, Majumder A, Fontes CMGA, et al (2009) Structural and biochemical properties of lichenase from *Clostridium thermocellum* Indian J Microbiol. 49: 72–76. 10.1007/s12088-009-0003-3 PMC3450042 23100753

[38] Gilead S, Shoham Y (1995) Purification and characterization of alpha-L-arabinofuranosidase from *Bacillus stearothermophilus* T-6 Appl Environ Microbiol. 61: 170–174. 10.1128/aem.61.1.170-174.1995 PMC167272 7887599

[39] Ahmed S, Charan R, Ghosh A, Goyal A (2012) Comparative modeling and ligand binding site prediction of a family 43 glycoside hydrolase from *Clostridium thermocellum* Journal of Proteins and Proteomics. 3: 31–38.

[40] de Sanctis D, Inácio JM, Lindley PF, de Sá-Nogueira I, Bento I (2013) New evidence for the role of calcium in the glycosidase reaction of GH43 arabinanases FEBS. 277: 4562–4574. 10.1111/j.1742-4658.2010.07870.x 20883454

[41] Moraïs S, Salama-Alber O, Barak Y, Hadar Y, Wilson DB, et al (2010) Functional association of catalytic and ancillary modules dictates enzymatic activity in glycoside hydrolase family 43 β-xylosidase J Biol Chem. 287: 9213–9221. 10.1074/jbc.M111.314286 PMC3308730 22270362

[42] Jordan DB, Lee CC, Wagschal K, Braker JD (2013) Activation of a GH43 β-xylosidase by divalent metal cations: slow binding of divalent metal and high substrate specificity Arch Biochem Biophys. 533: 79–87. 10.1016/j.abb.2013.02.020 23500142

[43] Finkelstein AV, Galzitskaya OV (2004) Physics of protein folding Phys Life Rev. 1: 23–56. 10.1016/j.plrev.2017.01.025 28190683

[44] McGuffin LJ, Bryson K, Jones DT (2000) The PSIPRED protein structure prediction server Bioinformatics. 15: 404–405. 10.1093/bioinformatics/16.4.404 10869041

[45] Pace CN, Scholtz JM (1998) A helix propensity scale based on experimental studies of peptides and proteins Biophys J. 75: 422–427. 10.1016/s0006-3495(98)77529-0 PMC1299714 9649402

[46] Fujiwara K, Toda H, Ikeguchi M (2012) Dependence of α-helical and β-sheet amino acid propensities on the overall protein fold type BMC Struct Biol. 12: 18. 10.1186/1472-6807-12-18 PMC3495713 22857400

